# Recombination-Mediated Genetic Engineering of a Bacterial Artificial Chromosome Clone of Modified Vaccinia virus Ankara (MVA)

**DOI:** 10.1371/journal.pone.0001638

**Published:** 2008-02-20

**Authors:** Matthew G. Cottingham, Rikke F. Andersen, Alexandra J. Spencer, Saroj Saurya, Julie Furze, Adrian V. S. Hill, Sarah C. Gilbert

**Affiliations:** Wellcome Trust Centre for Human Genetics and The Jenner Institute, University of Oxford, Oxford, United Kingdom; Cambridge University, United Kingdom

## Abstract

The production, manipulation and rescue of a bacterial artificial chromosome clone of Vaccinia virus (VAC-BAC) in order to expedite construction of expression vectors and mutagenesis of the genome has been described (Domi & Moss, 2002, *PNAS* **99** 12415–20). The genomic BAC clone was ‘rescued’ back to infectious virus using a Fowlpox virus helper to supply transcriptional machinery. We apply here a similar approach to the attenuated strain Modified Vaccinia virus Ankara (MVA), now widely used as a safe non-replicating recombinant vaccine vector in mammals, including humans. Four apparently full-length, rescuable clones were obtained, which had indistinguishable immunogenicity in mice. One clone was shotgun sequenced and found to be identical to the parent. We employed *GalK* recombination-mediated genetic engineering (recombineering) of MVA-BAC to delete five selected viral genes. Deletion of *C12L, A44L, A46R* or *B7R* did not significantly affect CD8^+^ T cell immunogenicity in BALB/c mice, but deletion of *B15R* enhanced specific CD8^+^ T cell responses to one of two endogenous viral epitopes (from the E2 and F2 proteins), in accordance with published work (Staib *et al.*, 2005, *J. Gen. Virol.* **86**, 1997–2006). In addition, we found a higher frequency of triple-positive IFN-γ, TNF-α and IL-2 secreting E3-specific CD8+ T-cells 8 weeks after vaccination with MVA lacking *B15R*. Furthermore, a recombinant vaccine capable of inducing CD8^+^ T cells against an epitope from *Plasmodium berghei* was created using *GalK* counterselection to insert an antigen expression cassette lacking a tandem marker gene into the traditional thymidine kinase locus of MVA-BAC. MVA continues to feature prominently in clinical trials of recombinant vaccines against diseases such as HIV-AIDS, malaria and tuberculosis. Here we demonstrate in proof-of-concept experiments that MVA-BAC recombineering is a viable route to more rapid and efficient generation of new candidate mutant and recombinant vaccines based on a clinically deployable viral vector.

## Introduction

Modified Vaccinia virus Ankara (MVA) is a replication-deficient attenuated poxvirus that was derived from Vaccinia virus by Mayr *et al.* through more than 500 blind passages in chick embryo fibroblast (CEF) cell culture [1]. With the notable exception of the Syrian hamster cell line BHK-21 [2], MVA is unable to replicate productively in mammalian cells, though genome replication, late gene expression and immature virion formation usually occur [3], [4]. During its attenuation, MVA acquired large genomic deletions totalling about 30 kb, resulting in the complete loss of 26 open reading frames (ORFs), together with truncation or fragmentation of a further 21 ORFs and numerous smaller scale mutations [5]. MVA does not therefore express many of the known poxviral immune evasion and virulence factors [6]–[8], though how this determines its abortive phenotype in mammalian cells is not well understood [9]. Despite these features, MVA is at least as immunogenic as conventional replicating Vaccinia virus [10]–[13] and has the advantage of a considerably improved safety profile [14].

Attention has focussed on MVA not only as a possible ‘next generation’ smallpox vaccine [15]–[17], but also as a recombinant vaccine vector, with particular value in combination with other vectors (e.g. DNA, adenovirus, or an avian poxvirus) as a component of heterologous prime-boost regimens designed to elicit high frequencies of antigen-specific T cells [18]. This approach is undergoing development and clinical trial for diseases such as HIV-AIDS [19], [20], tuberculosis [21], [22] and malaria [23], [24], where protective T cell responses are required for vaccine efficacy. In the case of liver-stage malaria vaccines, specific cytotoxic T cell responses correlate with the limited levels of protection achieved so far in humans [25], but current vectors and regimens are not immunogenic enough to achieve significant efficacy in malarious regions [26]. There is therefore a need to identify recombinant vaccine vectors with greater immunogenicity, either by using novel vectors (e.g. simian adenoviruses [27]) or by improving existing platforms.

The routes available for attempts to improve MVA vector immunogenicity can be crudely divided into addition or removal of genes. Co-expression of costimulatory molecules, such as B7.1 [28] and 4-1BB ligand [29] and various cytokines, including IL-12 [30], IL-15 [31] and GM-CSF [32] have been reported to increase recombinant poxvirus vaccine immunogenicity. These interventions can achieve a two- to four-fold increase in peak murine IFN-γ T cell responses. The parallel strategy is to delete poxviral genes encoding immunomodulators that attenuate adaptive immune responses, but are not required for growth *in vitro*. During attenuation MVA lost many such factors, including soluble decoy receptors for IFN-γ [33], TNF-α [34], IFN-α/β [35] and various chemokines [36]; an inhibitor of IL-1β converting enzyme [37], the complement control protein [38] and an intracellular inhibitor of Toll-like receptor (TLR) and IL-1 receptor (IL-1R) signalling [39]. On the other hand, it retains genes encoding secreted interleukin- [40]–[42] and chemokine- [36], [43] binding proteins; a dehydrogenase involved in steroid synthesis [44], [45], a second inhibitor of TLR/IL-1 receptor signalling [46], and other genes implicated in poxviral virulence [47]–[50]. Evidence that deletion of such genes provides a route for improvement of MVA immunogenicity is provided by reports of augmented protective mouse CD8^+^ T cell responses elicited by MVA lacking *B15R*, encoding an IL-1β binding protein [51], and *A41L*, encoding a chemokine binding protein of unknown specificity [43].

In addition to *a priori* candidates, there are at least 30 MVA genes whose function remains unknown. Although many poxviral proteins retain sequence homology and functional similarity with host proteins, it has recently been shown in the case of Vaccinia virus protein N1 that preservation of structure and function can occur in the absence of significant sequence similarity [52]. An unbiased experimental approach is therefore required for identification of MVA genes that negatively affect its immunogenicity. In order to expedite this approach, we have constructed a bacterial artificial chromosome (BAC) clone of the MVA genome using the elegant method devised by A. Domi and B. Moss for Vaccinia virus [53], with slight modifications. Recombination-mediated genetic engineering (recombineering) of this construct [54], [55] permits more rapid generation of mutants for analysis than can be achieved by traditional methods, with the possible exception of host-range selection on rabbit cells using *K1L* [56], [57]. To test this concept, we deleted five MVA genes using recombineering of a fully sequenced BAC clone, rescued the constructs to infectious virus, and here demonstrate a modest but significant improvement in immunogenicity of MVA lacking *B15R*, supporting and extending published work [51]. For large-scale biomanufacturing of an MVA-based vaccine for clinical use, the ability to use clonal purifed BAC DNA as the starting-point, rather than a conventional plaque-purified recombinant, is likely to be of considerable value. With this aim in mind, as well as pre-clinical studies, we demonstrate immunogenicity of a recombinant antigen inserted at the traditional thymidine kinase locus of MVA-BAC by recombineering.

## Materials and Methods

### Recombinant MVA construction

**Figure 1:**
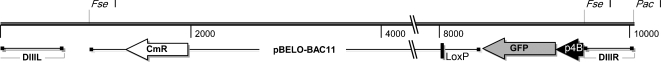
Schematic of construct for recombination into deletion III locus of MVA, based on pBELO-BAC11. DIIIL and DIIIR = left and right flanks for homologous recombination into MVA genome; GFP = green fluorescent protein gene; p4B = Fowlpox virus late promoter; CmR = chloramphenicol acetyltransferase gene for selection in *E. coli*. Flanking *Fse*I sites are absent from MVA and permit excision of the cassette. The *Pac*I site is provided for linearisation of the plasmid prior to recombination with MVA in infected transfected cells.

Figure 1 shows the plasmid construct used to generate a recombinant virus (referred to as MVA-BAC-parent) containing the entire sequence of the BAC vector pBELO-BAC11 at the Deletion III locus of MVA (between the remnants of *A51R* and *A56R*) together with a GFP reporter gene driven by the p4B late promoter from Fowlpox virus. The fragments required were amplified by PCR with primers designed to introduce restriction sites for assembly by standard methods. The flanking regions for recombination correspond to positions 148412-149083 and 149340-149855 of MVA genome sequence U94848 [5]. The plasmid was linearised with *Pac*I and transfected into MVA-infected primary chick embryo fibroblast (CEF) cells (Institute for Animal Health, Compton, UK). The cells were trypsinised and GFP-positive cells were sorted by flow cytometry on a Dako Cytomation MoFlo prior to plaque picking of the recombinant virus to purity, amplification and titration on CEFs. Cells were maintained in Dulbecco's modified Eagle medium (DMEM) supplemented with 10% FCS or with 2% FCS for viral growth. Identity and purity were confirmed by PCR. MVA expressing the recombinant antigen TIP was prepared as described (A. Spencer *et al.*, manuscript in preparation).

### Generation of MVA-BAC

CEF cells in 6-well plates were infected with MVA-BAC-parent at 5 pfu/cell and 2 h later the inoculum was replaced with growth medium containing 45 µM isatin-β-thiosemicarbazone (IβT). The compound was kindly synthesised by Dr J. Robertson (Chemistry Research Laboratory, Oxford University) and was dissolved to 5 mg/mL in acetone for storage at −20°C, then diluted to 1mg/mL in 0.25 M NaOH immediately prior to use. The cells in some wells were transfected with pCI-Cre (obtained from A. Domi, NIAID, NIH, Bethesda, MD) using Lipofectamine (Invitrogen). The DNA was phenol-extracted from the cultures 24h later as described [53] and resuspended in 20 µl of Tris-EDTA. Of this material, 3 µl was electroporated into DH10B *E. coli* (Invitrogen) prior to selection on LB plates with chloramphenicol (12.5 µg/mL) and miniprep by alkaline lysis from liquid LB cultures. Clones were screened by PCR for the presence of the genes *MVA005, MVA010, MVA044, MVA086* and *MVA188.* For restriction mapping and pulsed-field gel electrophoresis, BAC DNA was prepped by QIAgen kit and 0.5–1 µg of DNA was digested overnight in a 15 µl reaction with the relevant enzymes (NEB). MVA-BAC clone 26 was shotgun sequenced at eightfold coverage by Lark Technologies (Cogenics), Inc.

### BAC rescue

BHK-21 cells (maintained in DMEM+10% FCS) were seeded into 6-well plates and infected with FP9-LacZ [58], an attenuated strain of Fowlpox virus expressing β-galactosidase, at 1 pfu/cell in Optimem (Gibco). After 1–2 hours, the cells were transfected with 4 µg of Qiagen-purified MVA-BAC DNA using Lipofectamine 2000 (Invitrogen) in Optimem. The BAC DNA was stored at 4°C, was never vortexed, and was pipetted only with cut-off tips. The cells were monitored for GFP expression by epifluorescence microscopy and were harvested by freeze-thaw 4–6 days later. The lysate was used to infect fresh BHK cell monolayers and the cultures were monitored for the appearance of virus growth. Rescued viruses were amplified in BHK cells using DMEM without serum for infection and growth, before purification over sucrose cushions and titration on CEFs.

### BAC recombineering

*GalK*-based recombineering was done exactly as described by S. Warming *et al.* [55], from whom the reagents and strains were obtained (see also http://recombineering.ncifcrf.gov/). Sequencing of PCR products and primer synthesis was performed by MWG-Biotech AG.

### Mouse immunogenicity

Female BALB/c and C57BL/6 mice aged 6 to 8 weeks were obtained from the Biomedical Services Unit, Oxford University and procedures were conducted according to the UK Animals (Scientific Procedures) Act 1986. Mice were anaesthetised with ketamine-dormitor prior to intradermal immunisation with 10^6^ pfu of MVA divided into two 25 µl injections (one per ear). Splenocytes were harvested as described [58] seven days post-immunisation for flow cytometric analysis or fourteen days post-immunisation for ELIspot analysis. ELIspots were conducted using 18–20 h stimulation with 1 µg/mL synthetic peptide (ProImmune) in IVPH plates (Millipore) coated with anti-IFN-γ antibody AN18 (Mabtech). Spots were developed with R46A2-biotin (Mabtech) followed by streptavidin alkaline phosphatase (Mabtech) and substrate kit (BioRad) and enumerated on an automated reader (Autoimmun Diagnostika). For flow cytometry, cells were stimulated for 6h with 1 µg/mL peptide in the presence of 2 µl/mL Golgi-Plug (BD) and stained with anti-CD8 pacific blue conjugate and anti-CD4 APC-Alexa-750 conjugate prior to fixation in 10% neutral buffered formalin (Sigma). Intracellular cytokine staining was performed using FITC-conjugated anti TNF-α, PE-conjugated anti-IL-2 and Alexa-647-conjugated anti-IFN-γ diluted in Cytoperm (BD). All antibodies were obtained from eBiosciences. Data were acquired on a CyAn flow cytometer (Dako) and analysed using FlowJo (Treestar). PESTLE and SPICE software were obtained from Dr M. Roederer, Vaccine Research Centre, NIH. Other statistical analyses were performed using Prism (GraphPad Software, Inc.).

## Results

### Cloning of MVA genome into BAC

A procedure very similar to that developed by Domi and Moss for VAC-BAC [53] was used to create BACs containing the genome of MVA. First, a cassette containing pBELO-BAC11, a LoxP recombinase site and a GFP reporter gene driven by a late poxviral promoter (see Figure 1) was inserted into the deletion III locus of MVA by conventional recombination in infected CEF cells. Cells infected with this recombinant virus, referred to at MVA-BAC-parent, were treated with isatin-β-thiosemicarbazone (IβT) to inhibit viral hairpin resolution and promote genome concatemerisation [59]. After transformation of *E. coli* with DNA from these cells, dozens of chloramphenicol-resistant colonies were observed regardless of treatment of infected cells with IβT. Unexpectedly, transfection of cells with pCI-Cre prior to infection in an attempt to induce recombination between LoxP sites to produce circular molecules from head-to-tail concatemers [53] dramatically decreased the number of colonies obtained, probably due to cytotoxicity of the transfection reagent. Alternatively, it is possible that cryptic LoxP sites such as those present in mammalian genomes [60] resulted in illegitimate recombination events.

Colonies were screened for potential full-length clones by PCR at five loci spaced along the genome, and candidates were further characterised by restriction mapping with *Hin*dIII and *Xho*I and pulsed-field gel electrophoresis after digestion with *Fse*I to excise the BAC cassette (data not shown). No full-length clones were isolated from pCI-Cre transfected samples (of 5 colonies screened), but from untransfected cells, four apparently full-length clones were obtained, three derived from IβT-treated cells (of 24 colonies screened) and one from untreated cells (of 18 colonies screened). This frequency is very similar to that observed for VAC-BAC [53].

### BAC rescue

The next step was to “rescue” the BAC clones to infectious MVA using a Fowlpox virus helper to provide transcriptional machinery. The BHK cell line, which is the only mammalian cell line known to support MVA replication, is ideal for this purpose since it is non-permissive for the host-range restricted avipoxviruses. Following transfection into BHK cells infected with Fowlpox virus, all four of the MVA-BAC clones were converted to infectious MVA able to replicate and form plaques on BHK and CEF cells. We have not conducted formal growth rate analysis of the resulting viruses, but viral titres of 8.0×10^8^ to 2.4×10^9^ pfu/mL (final volume ∼0.5 mL) were achieved following sucrose cushion purification from BHK cultures totalling 1500 cm^2^. The titrations were performed on CEF cells, in which Fowlpox virus can replicate, and absence of significant residual Fowlpox virus was demonstrated by X-Gal staining for the *LacZ* marker gene present in the helper virus.

### Immunogenicity of MVA-BACs

**Figure 2:**
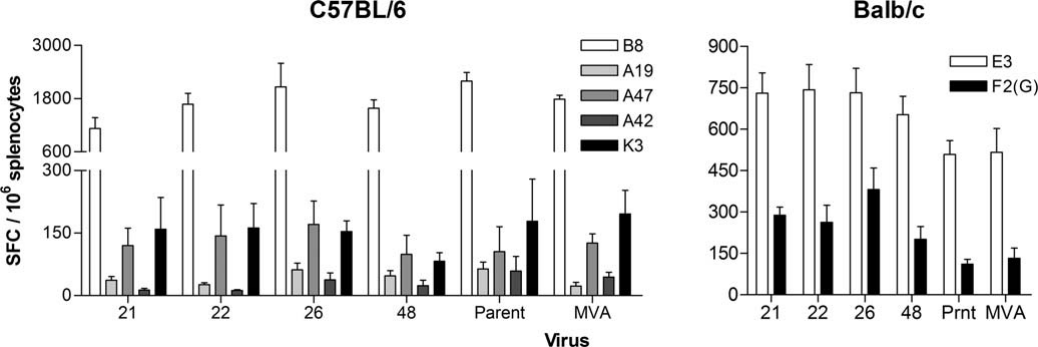
Immunogenicity of four MVA-BAC clones (21, 22, 26, 48) in C57BL/6 and BALB/c mice compared to parent virus (containing the BAC cassette) and MVA (lacking the BAC cassette). Mice were immunised with 10^6^ pfu i.d. and the specific T cell responses to the indicated endogenous synthetic peptide epitopes [61], [62] were measured 14 days later by IFN-γ ELIspot assay. Data are means and SEM from groups of four mice.

The principal utility of MVA is as a vaccine vector, particularly for eliciting T cell responses. The immunogenicity of the viruses derived from the four MVA-BAC clones was therefore assessed in mice by determining CD8^+^ T cell responses to viral antigens following intradermal inoculation. Tscharke *et al.* have described in detail the immunodominance hierarchy of CD8^+^ T cell “determinants” (i.e., epitopes) following infection of BALB/c and C57BL/6 mice with Vaccinia virus, MVA and other poxviruses [61], [62]. We used the described synthetic peptides to stimulate splenocytes from immunised BALB/c or C57BL/6 mice and quantified antigen-specific T cell responses by IFN-γ ELIspot assay (Figure 2).

Mice were immunised intradermally with the four MVA-BAC clones, the MVA-BAC-parent (from which the BAC clones were derived), or MVA (i.e. virus lacking the BAC cassette). In C57BL/6 mice, all were immunogenic and there was no statistically significant difference by one-way ANoVA between the T cell responses to any of these viruses for each of the five available determinants. In BALB/c mice, the same was true for the immunodominant determinant E3, but there were significant differences in the subdominant determinant specific F2(G) responses (p<0.01, one-way ANoVA), and a post-hoc test revealed that the statistically significant pairwise comparisons are clone #26 versus parent or MVA (p<0.05, Newman-Keuls multiple comparison test). Despite the lack of conventional significance for other clones, there is a trend towards higher responses to BAC-derived MVA in BALB/c mice, but this is not an effect of the presence of the BAC cassette at deletion III, since MVA-BAC-parent also contains this insertion.

The inability to distinguish the MVA-BAC clones by restriction map, viral growth yield or murine immunogenicity suggests that they are likely to be identical, but the possibility of some bias in the isolation procedure necessitates verification of clone fidelity by complete sequencing, especially prior to embarking on a programme of genetic manipulation.

### Sequence of MVA-BAC

Three MVA genome sequences are available in GenBank, one published by Antoine *et al.* [5] (U94848), one deposited by Bavarian Nordic GmbH (DQ983236) and the “Acambis 3000” strain (AY603355) deposited by the CDC. The Bavarian Nordic sequence is identical to the Acambis sequence, and these differ from Antoine *et al.*'s sequence only by five single nucleotide substitutions (excluding the repeat regions of the inverted terminal repeats, which are not fully described in the Acambis and Bavarian Nordic sequences). All of these loci are very polymorphic amongst strains of Vaccinia virus (http://www.poxvirus.org/). The mutations and genes affected are shown in Table 1.

**Table 1:** Polymorphisms in MVA and MVA-BAC clone #26.

Position^a^	Gene affected	Mutation	Coding change	MVA strains possessing mutation^b^	Example Vaccinia virus strain possessing mutation^c^
68740	*G6R*	A>G	N>D	A/BN, MVA-BAC	Lister, WR, TAN
73886	*L3L*	C>T	R>K	A/BN, MVA-BAC	WR
108489	*A4L*	C>T	Silent	A/BN, MVA-BAC	WR
114308	*A10L*	T>C	Silent	A/BN, MVA-BAC	WR
114945	*A10L*	C>T	R>K	A/BN, MVA-BAC	Lister
30326	*F7L*	6 bp ins.	NK ins.	MVA-BAC	TAN

a Refers to residue numbering of MVA genome sequence U94848 [5].

b A/BN = Acambis/Bavarian Nordic (see text).

c WR = Western Reserve; COP = Copenhagen; TAN = TanTien.

MVA-BAC clone #26 was selected for shotgun sequencing at eightfold coverage, resulting in a single contig (excluding the repeat regions), and was found to be identical to the Acambis/Bavarian Nordic strain, with the exception of a 6 bp insertion in the non-essential gene *F7L* [63]. The mutation codes for an extra Asn-Lys repeat and makes MVA-BAC identical to the TanTien strain of Vaccinia virus at this polymorphic locus, where other strains have two Asn-Lys repeats, with the exception of the Copenhagen strain which has eight (http://www.poxvirus.org/). In order to determine at what point in the genesis of MVA-BAC this mutation occurred, we sequenced the progenitor viruses at this locus by PCR amplification from genomic DNA. MVA-BAC-parent was found to possess the 6 bp insertion mutation, but its progenitor (from which the recombinant was derived) did not. Sequencing of PCR products revealed that a selection of other MVA recombinants from our laboratory and our original stock provided by Anton Mayr did not possess the mutation. It is not clear whether this 6 bp insertion arose during production of the MVA-BAC parent, or represents isolation of a rare genotype present at low levels in the source MVA. Evidence for genetic heterogeneity within poxvirus strains [64], [65](personal communication, R. Regnery) favours the latter possibility.

### Deletion of MVA genes by BAC recombineering

Recombination mediated genetic engineering (recombineering) permits modification of BAC DNA by homologous recombination in *E. coli*. We used the system described by Warming *et al.* [55], which is based on stringent temperature-sensitive expression of the λ Red genes *exo, bet* and *gam* coupled with *GalK*-based positive and negative selection. A similar approach, not using *GalK,* has been described for VAC-BAC by Domi and Moss [54], who also verified the stability of their Vaccinia virus BAC clone after induction of the λ Red system. Five genes reported to affect MVA immunogenicity or Vaccinia virus immunogenicity or virulence were selected from the literature for deletion by insertion of *GalK* in place of the ORF (see Table 2). Long oligonucleotide primers were used to introduce 50 bp homology arms at the 5′ and 3′ ends of *GalK* by PCR and these products were electroporated into heat-induced SW102 cells carrying MVA-BAC prior to selection on galactose minimal medium, as described [55]. Before rescue by transfection into Fowlpox virus infected BHK cells, as above, the modified BACs were checked by restriction digest with *Hin*dIII and *Xho*I and by sequencing two PCR products spanning the junctions at the *GalK* insertion. In only one case (the *A46R* deletion), was any mutation detected: a primer-derived single nucleotide deletion in the intergenic region downstream of the insertion. This mutation was ignored as it did not fall within any predicted regulatory sequences (promoters or terminators). *GalK* deletion mutant BACs were also checked for absence of undeleted contaminants by PCR across the deleted locus. All five modified *GalK*-carrying MVA-BACs converted into infectious virus, and again, though we did not formally compare growth rates, all were amplified to titres of 2.8×10^8^ to 2.6×10^9^ pfu/mL (final volume ∼0.5 mL) following sucrose cushion purification from 1500 cm^2^ of BHKs.

**Table 2:** MVA-BAC genes deleted by *GalK* recombineering.

Gene	Function of encoded protein	Refs	Left homology arm (bp)^a^	Right homology arm (bp)^a^	Mutations in MVA^b^
*C12L*	IL-18 binding protein	[41], [42]	13195-13224	13415-13464	6 bp in-frame deletion
*A44L*	Hydroxysteroid dehydrogenase	[44], [45]	142608-142657	143549-143598	1 amino acid substitution
*A46R*	Inhibitor of Toll-like receptor signalling	[46]	143941-143990	144699-144748	–
*B7R*	Chemokine binding protein (SCP-3^c^)	[36]	156989-157038	157564-157613	15 bp in-frame deletion
*B15R*	IL-1β binding protein	[51]	161971-162020	162999-163048	–

a Refers to residue numbering of MVA genome sequence U94848 [5].

b Coding mutations specific to MVA, i.e. not known in any other strain of Vaccinia virus.

c Smallpox virus encoded chemokine receptor (SECRET) domain containing protein 3.

### Immunogenicity of MVA-BAC deletion mutants

**Figure 3:**
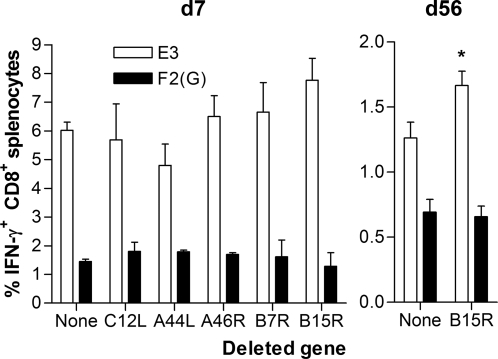
Peptide-specific splenic CD8^+^ T cell responses 7 days (d7) or 56 days (d56) after i.d. immunisation of BALB/c mice with 10^6^ pfu of MVA-BAC derived viruses, either unmodified or with indicated genes deleted by insertion of *GalK*. * p = 0.039 (*t*-test). Data are means and SEM from groups of four mice.

The effect of the above modifications on immunogenicity was assessed in intradermally immunised BALB/c mice. Peak specific T cell responses were analysed seven days later by flow cytometry with intracellular cytokine staining following stimulation with the F2(G) and E3 peptides (Figure 3). No statistically significant differences between groups were observed (one-way ANoVA for each epitope). Of the selected genes, only *B15R*, which encodes an IL-1β-binding protein, has been reported to affect MVA immunogenicity, rather than Vaccinia virus immunogenicity or virulence [51]. At the peak of the response, Staib *et al.* described a small non-significant effect of *B15R* deletion, very similar to that shown in Figure 3 for the E3 epitope, and with an identical p-value by *t*-test (p = 0.07); however, in the memory phase six months post-vaccination, they observed a highly significant ∼4.5-fold increase in CD8^+^ T cell response to the *B15R* deletion mutant. We therefore assessed memory responses at 8 weeks post-vaccination, and observed a small, but statistically significant increase in CD8^+^ T cell responses to E3 (p = 0.039, *t*-test), but not to F2(G). As well as the readout time, variation in dose (10^8^ vs. 10^6^ pfu), route (i.p. vs. i.d.), antigen (H3 vs. E3 peptide) and mouse strain (HHD vs. BALB/c) are likely to underlie the suggested difference in the magnitude of the effect of *B15R* deletion on memory T cell responses.

**Figure 4:**
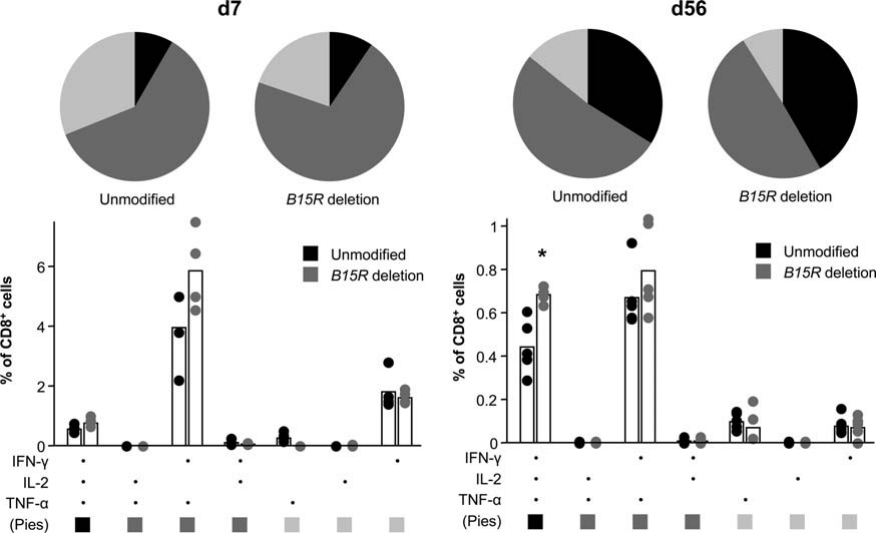
Multifunctionality of CD8^+^ T cells induced 7 days (d7) or 56 days (d56) after immunisation of BALB/c mice with unmodified MVA-BAC or *B15R* deletion mutant. Histograms show frequency of cells expressing each of the seven possible combinations of IFN-γ, IL-2 and TNF-α with bars showing the mean and circles showing the values for individual mice (n = 4). Pie charts show fraction of the total response comprised of cells expressing all three cytokines (black), any two cytokines (dark gray), or one cytokine only (light gray). * p = 0.018, Willcoxon-Rank test. Analysis performed using SPICE software [66].

In order to characterise the effect of *B15R* deletion further, we analysed E3-specific CD8^+^ T cell expression of TNF-α and IL-2 in addition to IFN-γ. SPICE software [66] was used to discriminate individual cells expressing the seven different ‘Boolean’ combinations of cytokines from the IFN-γ^+^, TNF-α^+^ and IL-2^+^ gated populations (Figure 4). This approach reveals that at the peak of the response (d7), most of the cells are IFN-γ and TNF-α double-positives. Furthermore, although there is no conventionally significant difference, the increase in bulk IFN-γ responses observed with the *B15R* deletion mutant is primarily the result of an increased number of these double-positive cells, rather than of the IFN-γ single-positive or the IFN-γ, TNF-α and IL-2 triple-positive cells that account for the remainder of the CD8^+^ T cell response. By contrast, at day 56, the effect of *B15R* deletion is seen to reside mainly within the triple-positive population, and the difference is statistically significant (p = 0.018, Willcoxon-Rank test). Deletion of *B15R* therefore affects both the quality and magnitude of the E3-specific CD8^+^ T cell response to MVA. Enhanced production of the mitogenic cytokine IL-2 during the early memory phase (8 weeks) may underpin the large difference in T cell frequency observed in Staib *et al.*'s 6 month experiment [51].

### Insertion of a recombinant antigen into MVA by BAC recombineering

In addition to more rapid production of deletion mutants, the MVA-BAC system would also have value as a faster method for insertion of genes encoding exogenous protective antigens or candidate ‘molecular adjuvants’ with the potential to increase vector immunogenicity. The elegance of the *GalK* based recombineering system [55] relies on the ability to counterselect for clones lacking *GalK* using deoxygalactose, which is metabolised to a toxic intermediate. This enables insertion of genes without the need for a selectable marker. However, loss of *GalK* can occur both by recombination with an electroporated DNA, or by deletion of a segment of the BAC. Unlike *GalK* insertion, which has an efficiency close to 100% in MVA-BAC, the specific replacement of *GalK* with an exogenous sequence has been achieved to date at a frequency of only up to 1% compared to spontaneous deletions.

**Figure 5:**
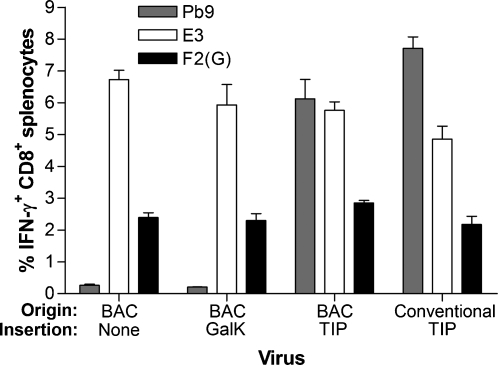
Immunogenicity of MVA-BAC expressing a recombinant antigen inserted at the standard thymidine kinase insertion site, in comparison to conventional recombinant MVA and recombineering precursors containing no insertion or *GalK*. The recombinant antigen, TIP, is an epitope string containing the Pb9 epitope from *Plasmodium berghei* circumsporozoite protein [67]. Bars show mean specific CD8^+^ T cell responses to the indicated peptides 7 days after i.d. vaccination of with 10^6^ pfu, from groups of four BALB/c mice, with error bars showing SEM.

We used this technique to insert an antigen expression cassette carrying no tandem reporter gene or selectable marker into the traditional thymidine kinase (TK) locus of MVA-BAC. This construct comprised the p7.5 early/late promoter driving an 82 amino acid epitope string, named “TIP” (for Tuberculosis, Immunodeficiency virus and Plasmodium), which contains relevant epitopes from various pathogens for vaccine-induced protection in mouse models, including the protective H-2K^d^-restricted Pb9 epitope from *Plasmodium berghei* circumsporozoite protein [67]. Figure 5 shows that Pb9-specific CD8^+^ T cell responses are elicited in MVA-BAC-TIP immunised mice to a level comparable with those elicited by conventional MVA-TIP. Although not statistically significant, there is a trend toward higher Pb9 responses (p = 0.07, *t*-test) and lower E3 responses (p = 0.11, *t*-test) in the conventionally derived MVA-TIP compared to the BAC-derived virus. This difference in immunodominance hierarchy may be attributable to differences in the nature of the cassette inserted at the TK locus (the lack of a late-promoter-driven GFP marker gene and opposite orientation of the TIP gene) or to the presence of the BAC cassette at deletion III.

## Discussion

Traditional methods for genetic manipulation of poxviruses, either for mutagenesis or expression of exogenous genes, rely on onerous plaque purification of rare recombinant viruses. Building on the results of Domi and Moss for VAC-BAC [53], [54], here we circumvent this requirement by creating an MVA-BAC that can be manipulated using recombineering and rescued to clonal viruses. With an efficiency similar to that observed in the case of VAC-BAC, we generated four MVA-BAC clones, which were identical by restriction map and had indistinguishable CD8^+^ T cell immunogenicity in two strains of mice. We sequenced one clone, and found it to be identical to published genomic sequences of MVA, with the exception of a minor mutation that is polymorphic amongst Vaccinia virus strains and was present in the parent MVA recombinant. We did not conduct an investigation into stability of the construct, since good stability had been shown for the even larger VAC-BAC construct [53], [54].

Of five candidate genes selected from the literature for deletion by MVA-BAC recombineering (Table 2), only one had any statistically significant effect on responses to viral CD8^+^ T cell epitopes following intradermal immunisation of BALB/c mice. The effect of *B15R* deletion was more pronounced 8 weeks post-vaccination, though not as great as was previously reported at a 6 month timepoint in HHD mice [51]. Although we did not observe an effect of similar magnitude using an earlier readout, we show using SPICE polychromatic flow cytometry analysis that antigen-specific CD8^+^ T cells from mice immunised with MVA lacking *B15R* are composed of a higher proportion of triple-positive cells that express IL-2 in addition to IFN-γ and TNF-α. This ‘multi-functional’ phenotype may be responsible for the large differences in cell frequency at 6-months post-vaccination [51], since IL-2 is strongly implicated in memory T cell homeostasis [68] and its expression by virus-specific CD8^+^ T cells has been reported to promote antigen-specific proliferation of these cells even in the absence of CD4^+^ T cell help [69]. Whether responses to a recombinant antigen will be augmented in a *B15R* deletion background remains to be tested, though it should be noted in this regard that enhancement of anti-vector responses was confined to the E3-specific response and was not observed in the case of the F2(G) epitope.

Deletion of four other genes by *GalK* insertion in MVA-BAC did not potentiate CD8^+^ T cell responses to the vector epitopes. *C12L, A44R* and *B7R* all carry MVA-specific mutations that do not occur in any other sequenced strain of Vaccinia virus. Although strain Ankara (from which MVA was derived) has not been sequenced, these mutations presumably arose during attenuation, and the genes affected may therefore already be inactive. Alternatively, or additionally, functional redundancy of families of immunomodulatory genes acting on the same pathways may negate the effect of the deletion mutants. Indeed, poxviruses appear to have taken a grapeshot-like approach to disruption of the TLR/IL-1R signalling pathway [6], though MVA's gun has been spiked by deletion of many components, including *A52R*, which like *A46R* can inhibit NF-κB activation, albeit by a distinct subset of stimuli [46].

The deletion of these genes also did not impair the CD8^+^ T cell responses to the vector, suggesting that, as well as being dispensable for growth in BHK cell culture, they are not required for circumvention of host defence mechanisms operating *in vivo* (though since MVA is non-replicating in mammals, this conclusion does not extend to poxviral pathogenesis). If multiple MVA genes were to be deleted, in order to overcome putative functional redundancy, one might assume that at some point the virus would be so crippled as to be only very poorly immunogenic, for example by an antiviral response occurring early during the infection cycle, which normally proceeds to immature virion formation even in non-permissive cells. On the other hand, the major evolutionary driving force for acquisition of immunomodulators by poxviruses is more likely productive replication of a virulent virus, rather than evasion of adaptive responses (*i.e*. antibody or cytotoxic T cell responses). The MVA-BAC recombineering system will hopefully be versatile and rapid enough to allow these subtleties to be addressed, especially as it is likely to speed up the production of ‘revertant’ viruses in order to confirm the genetic specificity of a novel phenotype. Furthermore, the ability to make multiple modifications by ‘recycling’ the *GalK* dual-selectable marker is likely to be of particular value.

Other applications of MVA-BAC include production of viruses carrying recombinant antigens and/or molecular adjuvants for pre-clinical evaluation and clinical trial. The use of BAC DNA and an inactivated clinical grade helper virus as the input to a manufacturing process could reduce the burden of traceability currently required by regulatory authorities, though a method for efficient removal of the BAC cassette would be needed (see Figure 1). In this setting, the issues of construct stability and helper virus contamination would doubtless require more rigorous investigation than described here. Insertion of antigen expression cassettes by recombineering additionally circumvents the requirement for a reporter gene or selectable marker and its removal by transient-dominant selection [70] when a markerless product is required. In this regard, the difficulty of insertion of the TIP antigen by *GalK* counterselection is rather disappointing, though there is very likely room for improvement, for example by using longer homology arms in the targeting DNA, or electroporating maximal quantities of DNA. When removing *GalK* without concomitant insertion of DNA (i.e., from a deletion locus), we have achieved much higher recombineering efficiencies of up to 95% (data not shown). Alternatively, recombination in RecA^+^ *E. coli* [71] is a potential means of inserting elements without the use of a selectable marker, since several rare restriction sites (required for linearisation of the BAC prior to recombination) are absent from the MVA genome. For pre-clinical work, an *in vitro* site specific recombination system could be developed [72].

The vaccine vector capability of Vaccinia virus expressing a recombinant antigen was demonstrated nearly 25 years ago [73], [74]. Successful vectored vaccines for diseases such as malaria, HIV-AIDS and tuberculosis will likely require combinations of antigens and delivery systems able to induce immune responses of the right character and magnitude against the recombinant antigens. Currently, MVA is one of the more promising deployable delivery systems and has potential for improvement. The MVA-BAC technology provides an opportunity to accelerate production of candidate vaccines for evaluation, and potentially a superior starting point for clinical vaccine manufacture.
