# Correction: Recombination-Mediated Genetic Engineering of a Bacterial Artificial Chromosome Clone of Modified Vaccinia virus Ankara (MVA)

**DOI:** 10.1371/annotation/2b1d4029-f72a-4fa9-9776-20e4073f650e

**Published:** 2011-02-26

**Authors:** Matthew G. Cottingham, Rikke F. Andersen, Alexandra J. Spencer, Saroj Saurya, Julie Furze, Adrian V. S. Hill, Sarah C. Gilbert

The following information was missing from the Funding section: "The study was supported by funding from the NIHR Oxford Biomedical Research Centre programme."

